# Fe–N–C catalyst with atomic Fe dispersion and hierarchical porosity *via* PVP-assisted MOF synthesis for ORR in acidic media

**DOI:** 10.1039/d5ra05653e

**Published:** 2025-09-22

**Authors:** Muhammad Shoaib, Shagufta Ishtiaque, Ahsan Abdul Ghani, Zahoor Awan, Abid Ali, Ayisha Rubab, Maaz Akhtar

**Affiliations:** a Department of Chemical Engineering, University of Karachi Karachi Pakistan ahsan.ghani@uok.edu.pk; b Department of Food Engineering, NED University of Engineering and Technology Karachi Pakistan; c Chongqing Key Laboratory for Advanced Materials and Technologies of Clean Energies, School of Materials and Energy, Southwest University Chongqing 400715 PR China; d Industrial Engineering Department, College of Engineering, Imam Mohammad Ibn Saud Islamic University (IMSIU) Riyadh Kingdom of Saudi Arabia

## Abstract

Iron–nitrogen–carbon (Fe–N–C) catalysts have emerged as leading non-precious metal catalyst (NPMC) candidates for the oxygen reduction reaction (ORR) in acidic media, yet challenges persist in achieving high activity, durability, and atomic-scale Fe dispersion. Here, we report Fe-ZIF-8-PVP-1000, a high-performance Fe–N–C catalyst synthesized *via* a polyvinylpyrrolidone (PVP)-assisted metal–organic framework (MOF) strategy. PVP functions as a morphology stabilizer, nitrogen dopant, and metal dispersant, producing atomically dispersed Fe–N_*x*_ sites within a hierarchically porous, nitrogen-rich carbon matrix. The catalyst achieves a half-wave potential (*E*_1/2_) of 0.865 V *vs.* RHE in 0.5 M H_2_SO_4_, surpassing commercial 28.6 wt% Pt/C (*E*_1/2_ ≈ 0.855 V) and rivaling the best Fe–N–C catalysts reported to date. With a BET surface area of 1579.8 m^2^ g^−1^ and a micropore volume of 0.54 cm^3^ g^−1^, it provides abundant accessible active sites; electrochemical analysis further revealed an electron transfer number of *n* ≈ 3.96 with minimal H_2_O_2_ yield, confirming a highly selective four-electron ORR pathway. The catalyst retained approximately 84% of its current after 10 h and exhibited only an 11 mV negative shift in *E*_1/2_ after 30 000 cycles, demonstrating outstanding stability. This scalable synthesis provides acid-tolerant, high-performance NPMCs with optimized structural and electronic properties.

## Abstract 2

A PVP-assisted MOF strategy integrates atomic Fe dispersion, Fe–N_*x*_ enrichment, and porosity in one step, yielding a durable Fe–N–C catalyst that surpasses Pt/C in acidic ORR.

## Graphical abstract

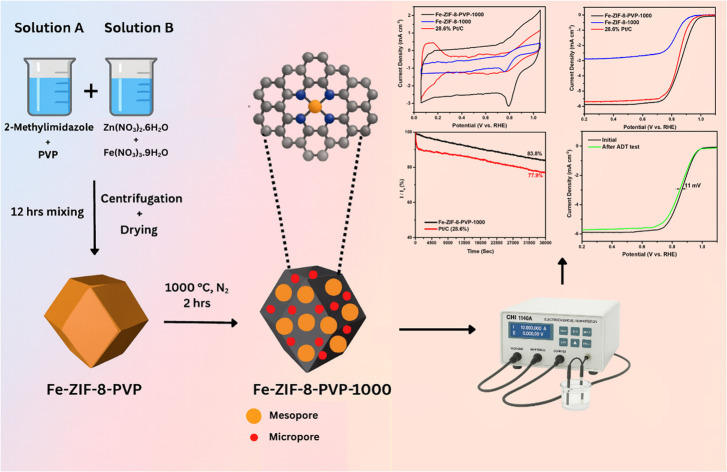

## 1 Introduction

The oxygen reduction reaction (ORR) remains a critical kinetic bottleneck in the advancement of fuel cells, particularly proton exchange membrane fuel cells (PEMFCs),^1,2^ which are central to the decarbonization of transportation and other energy-intensive sectors. While platinum-based catalysts exhibit outstanding ORR activity, their widespread application is hindered by high cost, limited availability, and the high environmental footprint associated with platinum extraction and processing.^3,4^ These limitations have spurred extensive research into non-precious metal catalysts (NPMCs) capable of delivering comparable performance under the harsh electrochemical conditions typical of acidic fuel cell environments. Iron–nitrogen–carbon (Fe–N–C) catalysts have emerged as the most promising class of NPMCs for the oxygen reduction reaction (ORR), offering a compelling combination of cost-effectiveness, tunability, and environmental sustainability.^5–14^

A transformative development in this field has been the use of metal–organic frameworks (MOFs), particularly zeolitic imidazolate frameworks (ZIFs), as precursors. These frameworks provide highly tunable, nitrogen-rich porous scaffolds that can incorporate Fe species and direct the formation of dispersed Fe–N_*x*_ motifs upon pyrolysis,^15–20^ which are widely recognized as the primary active sites for ORR in acidic media.^21–28^ Among them, ZIF-8 has emerged as the most widely employed template,^29–35^ and ZIF-8-derived Fe–N–C catalysts—obtained by introducing Fe into ZIF-8 through methods such as ion exchange, impregnation, or gas-phase infiltration—have proven effective in yielding carbonized structures enriched with Fe–N_4_ active sites.^36,37^ For example, Wan *et al.*^38^ created concave-structured, ZIF-8-derived Fe–N–C catalysts with improved Fe–N_4_ utilization; Jiao *et al.*^39^ anchored Fe–N_4_ only at accessible sites *via* chemical vapor deposition, achieving nearly full utilization; and Mehmood *et al.*^40^ reached ultra-high Fe loading (∼7 wt%) with atomically dispersed Fe–N_4_, quantitatively linking site density and turnover frequency to PEMFC activity. Together, these studies establish a versatile platform for developing Fe single-atom catalysts, where both site density and accessibility govern performance.

Porosity engineering has also emerged as a decisive factor for maximizing site utilization. Zhou *et al.*^41^ enhanced microporosity by using melamine-assisted ZIF-8, which increased the density of accessible sites; Guo *et al.*^42^ employed covalent–organic–polymer-derived Fe–N–C with hierarchical pores, showing excellent performance in PEMFCs despite lower RDE activity; and a recent report demonstrated that tuning the growth temperature of ZIF-8 precursors produced particles with integrated micro/mesoporosity, resulting in exceptional site utilization.^43^ Collectively, these findings highlight that hierarchical porosity is as crucial as Fe–N_4_ formation, since it dictates both mass-transport efficiency and the fraction of electrochemically active sites.^44–47^

Despite this progress, Fe–N–C catalysts in acidic media still face two critical limitations: (i) Fe precursors aggregate into nanoparticles during high-temperature pyrolysis, creating non-uniform and unstable sites, and (ii) the ZIF-8 framework often collapses, reducing microporosity and hindering accessibility.^48–51^ Overcoming these challenges requires precise chemical control during synthesis and carbonization. One increasingly adopted strategy is the incorporation of polyvinylpyrrolidone (PVP), an amphiphilic polymer capable of interacting with both metal ions and MOF precursors.^52–54^ The carbonyl and pyrrolidone groups of PVP chelate Fe ions, preventing aggregation and ensuring their homogeneous distribution during ZIF-8 crystallization.^55,56^ Its steric hindrance regulates crystal nucleation and growth, yielding more uniform Fe-ZIF-8 particles while suppressing collapse during pyrolysis.^53,57^ Upon carbonization, PVP provides an additional nitrogen-rich source, generating pyridinic and graphitic N that stabilize Fe–N_4_ moieties and improve conductivity. Furthermore, a PVP-derived coating around ZIF-8 particles preserves microporosity and prevents Fe sintering at high temperatures, thereby increasing site accessibility.^53,55–57^ Representative studies have confirmed these roles: Wang *et al.* demonstrated that PVP regulates Fe–N_4_ formation and conductivity in NaCl-templated catalysts;^58^ Yang *et al.* used a Fe(iii)–tannic acid–PVP complex to create atomically dispersed Fe–N_*x*_ with enhanced bifunctional activity;^59^ Wang *et al.* demonstrated that PVP coordinates Fe ions during ZIF-8 growth, improving site utilization;^60^ and Yue *et al.* reported PVP-coated ZIF-8 particles that confined Fe during pyrolysis and preserved porosity.^61^ However, these studies primarily emphasized alkaline ORR and did not address the combined challenge of atomic dispersion and structural integrity under acidic conditions.^49,62,63^

Here, we explicitly target these two limitations in acidic media by presenting Fe-ZIF-8-PVP-1000, a PVP-assisted Fe–N–C catalyst that overcomes Fe aggregation and microporosity loss during pyrolysis, thereby achieving atomic Fe dispersion, enhanced Fe–N_*x*_ site formation, and hierarchical porosity in a single step. In our approach, PVP coordinates Fe ions and regulates ZIF-8 growth, while also serving as an additional nitrogen dopant that enriches Fe–N_*x*_ site density and improves conductivity. Consequently, we attain a remarkable half-wave potential of 0.865 V (*vs.* RHE) in 0.5 M H_2_SO_4_, significantly surpassing the performance of the benchmark Pt/C (28.6 wt% Pt, *E*_1_/_2_ ≈ 0.855 V) under identical conditions, and achieving activity among the highest reported for Fe–N–C-based ORR catalysts in acidic media. Equally important, exceptional electrochemical stability was observed, as the catalyst preserved approximately 84% of its original ORR current following 10 hours of continuous operation, compared to the markedly quicker decline of Pt/C. This establishes a rational pathway for designing acid-tolerant Fe–N–C catalysts by coupling molecular-level site stabilization, Fe–N_*x*_ enrichment, and architecture-level mass-transport optimization.

## 2 Experimental

### 2.1 Catalyst preparation

1.000 g of 2-methylimidazole (MeIm, C_4_H_6_N_2_) was dissolved in 62.5 mL methanol to form Solution A. Separately, 0.3125 g of polyvinylpyrrolidone (PVP) was added to Solution A under stirring until completely dissolved. In parallel, Solution B was prepared by dissolving 0.600 g of zinc nitrate hexahydrate (Zn(NO_3_)_2_·6H_2_O) and 0.026 g of iron(iii) nitrate nonahydrate (Fe(NO_3_)_3_·9H_2_O) in 62.5 mL methanol. Solution B was then rapidly poured into Solution A under vigorous stirring. The mixture was stirred at room temperature for 12 h, during which PVP-coordinated Fe-doped ZIF-8 crystals formed. The solid product was collected by centrifugation (7200 rpm for 10 min) and washed with ethanol three times to remove unreacted species and excess polymer. After vacuum filtration, the powder was dried at 60 °C for 8 h in a vacuum oven, yielding the Fe-ZIF-8-PVP precursor. To obtain the catalyst, the dry precursor was pyrolyzed in a tube furnace at 1000 °C for 2 h under flowing N_2_ (heating ramp 5 °C min^−1^). The resulting black carbonaceous solid is denoted Fe-ZIF-8-PVP-1000. For the non-PVP counterpart (Fe-ZIF-8-1000), the same procedure was followed except that no PVP was added to Solution A; the Fe and Zn precursors were identical.

### 2.2 Characterization and electrochemical measurement

PANalytical X'Pert PRO diffractometer utilizing Cu Kα radiation (*λ* = 1.5406 Å) was employed to record the XRD patterns over 10–80° at 4° min^−1^. Morphological features were analyzed by FESEM (Zeiss Ultra55, 5 kV) and TEM (Hitachi H-9500, 300 kV). Quantachrome Autosorb-1-C instrument was used to collect N_2_ adsorption–desorption isotherms at 77 K after degassing at 200 °C for 2 h. BET, BJH, t-plot, DR, and DFT models were applied to determine surface area, pore size, and microporosity. XPS was conducted with a PHI-5000C (PerkinElmer, Mg Kα source), and spectra were analyzed using XPS Peak 4.1. ICP-OES was carried out on an Agilent 5900 after acid digestion of samples calcined at 650 °C.

Electrochemical characterization was carried out on a CHI 1140A workstation employing a conventional three-electrode system, in which a 3 mm glassy carbon electrode served as the working electrode, a saturated calomel electrode (SCE) acted as the reference,^64^ and a platinum wire was used as the counter electrode.^65–68^ The catalyst ink was prepared by ultrasonically dispersing 8 mg of sample in 3 mL ethanol and 0.2 mL 5 wt% Nafion solution. A 20 μL aliquot was drop-cast onto the glassy carbon electrode and dried at room temperature. All electrochemical measurements were carried out with a catalyst loading of 700 μg cm^−2^ (normalized to the geometric electrode area), while a loading of 60 μg cm^−2^ was used for the 28.6 wt% Pt/C catalyst. Cyclic voltammetry (CV) and linear sweep voltammetry (LSV) were recorded in O_2_-saturated 0.5 M H_2_SO_4_ at 25 °C, with a scan rate of 10 mV s^−1^. Potentials were converted to RHE, and electron transfer numbers were determined from RDE (BASi RDE-2) and Koutecky–Levich analysis. An accelerated durability test (ADT) was carried out by cycling the electrode between 0.6 and 1.0 V in O_2_-saturated electrolyte at 50 mV s^−1^ for 30 000 cycles under stationary conditions.^69–71^ LSVs were collected before and after the protocol at 10 mV s^−1^ with 1600 rpm rotation.^72^ The catalyst's stability was also verified by chronoamperometry at 0.8 V *versus* RHE in 0.5 M H_2_SO_4_ with continuous oxygen bubbling. Rotating ring-disk electrode (RRDE) tests were carried out using linear sweep voltammetry, sweeping the potential from 0.8 to 0.2 V at a rate of 5 mV s^−1^ with the electrode rotating at 1600 rpm.^73^ The H_2_O_2_ collection coefficient of the Pt ring was 0.37, based on calibration using the Fe(CN)_6_^4−/3−^ redox system. Further experimental details are provided in the SI.

## 3 Results and discussion

**Fig. 1:**
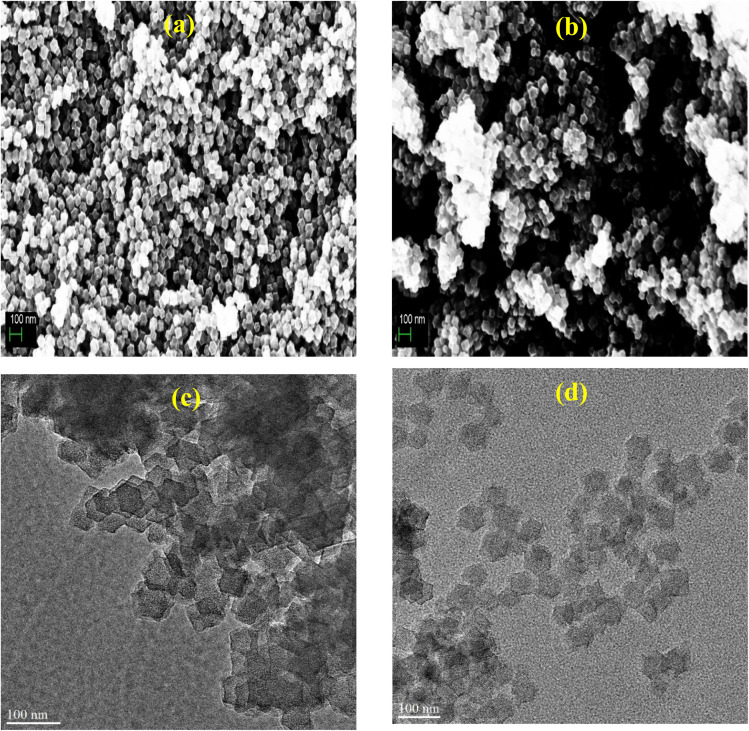
SEM images of (a) Fe-ZIF-8-PVP-1000 and (b) Fe-ZIF-8-1000, and TEM images of (c) Fe-ZIF-8-PVP-1000 and (d) Fe-ZIF-8-1000.

The morphology of Fe-ZIF-8 synthesized with and without polyvinylpyrrolidone (PVP) was examined using SEM, both before and after pyrolysis. Prior to thermal treatment, the PVP-modified sample displayed uniformly dispersed polyhedral crystals, typical of ZIF-8, whereas the material produced without PVP showed strong aggregation, forming large clusters of fused polyhedra (Fig. S1). These observations underscore the critical role of PVP as a stabilizing agent: its capping effect suppresses interparticle interactions, thereby enabling better control over crystal morphology. Following pyrolysis at 1000 °C, pronounced differences emerged between the two samples (Fig. 1a and b). The PVP-assisted material (Fe-ZIF-8-PVP-1000) largely preserved well-defined polyhedral structures with relatively smooth surfaces, reflecting the retention of ZIF-8-derived features under PVP protection. By contrast, the PVP-free material (Fe-ZIF-8-1000) exhibited agglomerated, partially collapsed particles with roughened textures, consistent with the absence of stabilization during carbonization. These observations align with previous reports demonstrating that PVP suppresses particle sintering and structural collapse during pyrolysis.^53,60^ TEM images (Fig. 1c and d) reveal that both Fe-ZIF-8-PVP-1000 and Fe-ZIF-8-1000 largely retain the polyhedral morphology characteristic of ZIF-8-derived materials,^74^ with comparable particle sizes of 50–60 nm. However, notable differences are observed in contrast, edge clarity, and internal texture. Fe-ZIF-8-PVP-1000 displays well-defined particles with smooth edges and uniform internal contrast, indicating preserved structure and homogeneously dispersed Fe species. The absence of dense or dark regions suggests minimal aggregation, supporting the role of PVP as a stabilizer that promotes atomic-level Fe dispersion.^75^ In comparison, Fe-ZIF-8-1000 shows slightly irregular particle edges and higher internal contrast in some domains, suggesting localized Fe enrichment or partial structural collapse. Although extensive nanoparticle aggregation is not observed, these features point to less uniform Fe incorporation in the absence of PVP.^60^

**Fig. 2:**
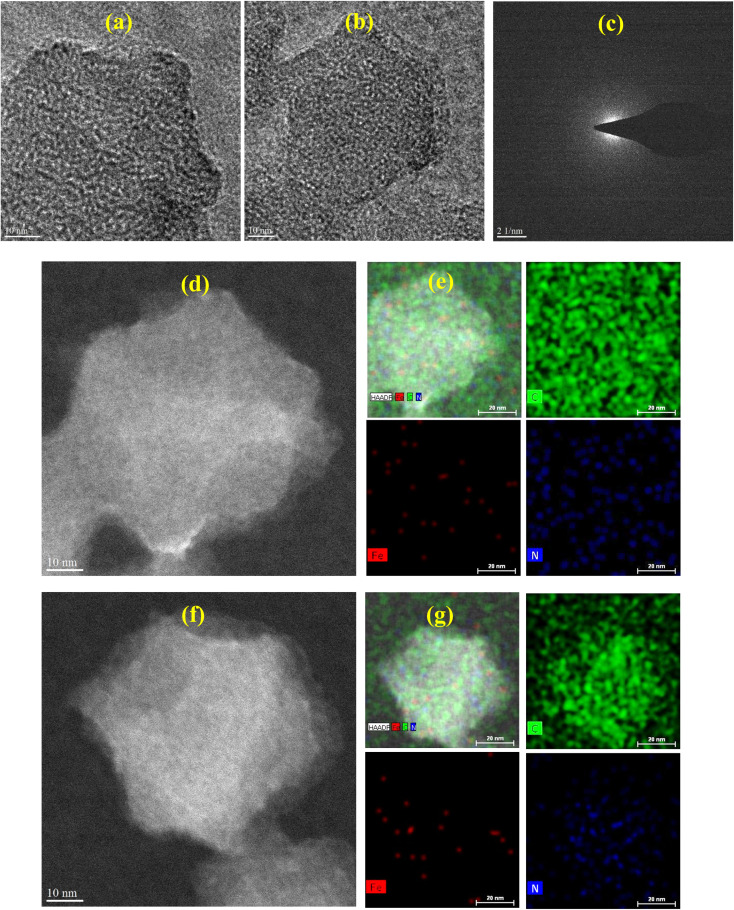
HRTEM images of (a) Fe-ZIF-8-PVP-1000 and (b) Fe-ZIF-8-1000. (c) SAED pattern of Fe-ZIF-8-PVP-1000. (d and e) HAADF-STEM image and corresponding EDX elemental mappings of Fe-ZIF-8-PVP-1000. (f and g) HAADF-STEM image and corresponding EDX elemental mappings of Fe-ZIF-8-1000.

HRTEM analyses (Fig. 2a and b) show that both samples exhibit amorphous carbon matrices with no visible lattice fringes or crystalline Fe domains, confirming the absence of large Fe particles and indicating atomic-scale dispersion.^76^ Fe-ZIF-8-PVP-1000 shows uniformly distributed, well-isolated bright spots, consistent with atomically dispersed Fe species.^77^ The homogeneous contrast and lack of clustering across different regions suggest that PVP effectively stabilizes Fe atoms during pyrolysis, preserving uniform dispersion. In contrast, Fe-ZIF-8-1000 displays a higher density of bright features with slight clustering and contrast variations, suggesting less uniform Fe distribution. These observations imply that without PVP, Fe tends to accumulate locally, although it remains in the sub-nanometer regime. Consistent with these HRTEM observations, the selected area electron diffraction (SAED) pattern of Fe-ZIF-8-PVP-1000 (Fig. 2c) displays diffuse diffraction rings, characteristic of a nanocrystalline structure embedded within an amorphous carbon matrix. The absence of sharp diffraction spots and the relatively broad ring pattern confirm that no large crystalline Fe or Fe_3_C domains are present,^76^ aligning with the HRTEM findings. However, the presence of very small nanoclusters below the detection limit of HRTEM and SAED cannot be entirely excluded. The combined evidence suggests that any Fe present is likely highly dispersed or exists as clusters too small to form long-range order, further validating the role of PVP in promoting atomic dispersion without forming larger crystalline domains.

HAADF-STEM imaging of Fe-ZIF-8-PVP-1000 (Fig. 2d) shows particles with smooth edges and uniform contrast, consistent with an even mass-thickness across the structure. The corresponding EDX maps (Fig. 2e) further demonstrate that the nitrogen content is not only clearly higher but also uniformly distributed, with Fe signals overlapping closely with both nitrogen and carbon. Such uniformity reflects the well-dispersed nature of Fe species and suggests favorable Fe–N_*x*_ coordination. The absence of Fe-rich clusters suggests the role of PVP in stabilizing isolated Fe atoms or small complexes during pyrolysis, thereby suppressing unwanted aggregation. In contrast, Fe-ZIF-8-1000 (Fig. 2f) displays sharper particle boundaries together with internal contrast variations, indicative of greater structural heterogeneity. The corresponding EDX maps (Fig. 2g) reveal Fe-rich domains, with a clear mismatch between Fe and N distributions. This non-uniform overlap points to partial aggregation of Fe and incomplete coordination with N. Although carbon remains broadly distributed in both catalysts, the weaker spatial correlation between Fe and N in Fe-ZIF-8-1000 highlights that, in the absence of PVP, Fe atoms are more prone to clustering and are consequently less effective at forming catalytically active Fe–N_*x*_ sites.

**Fig. 3:**
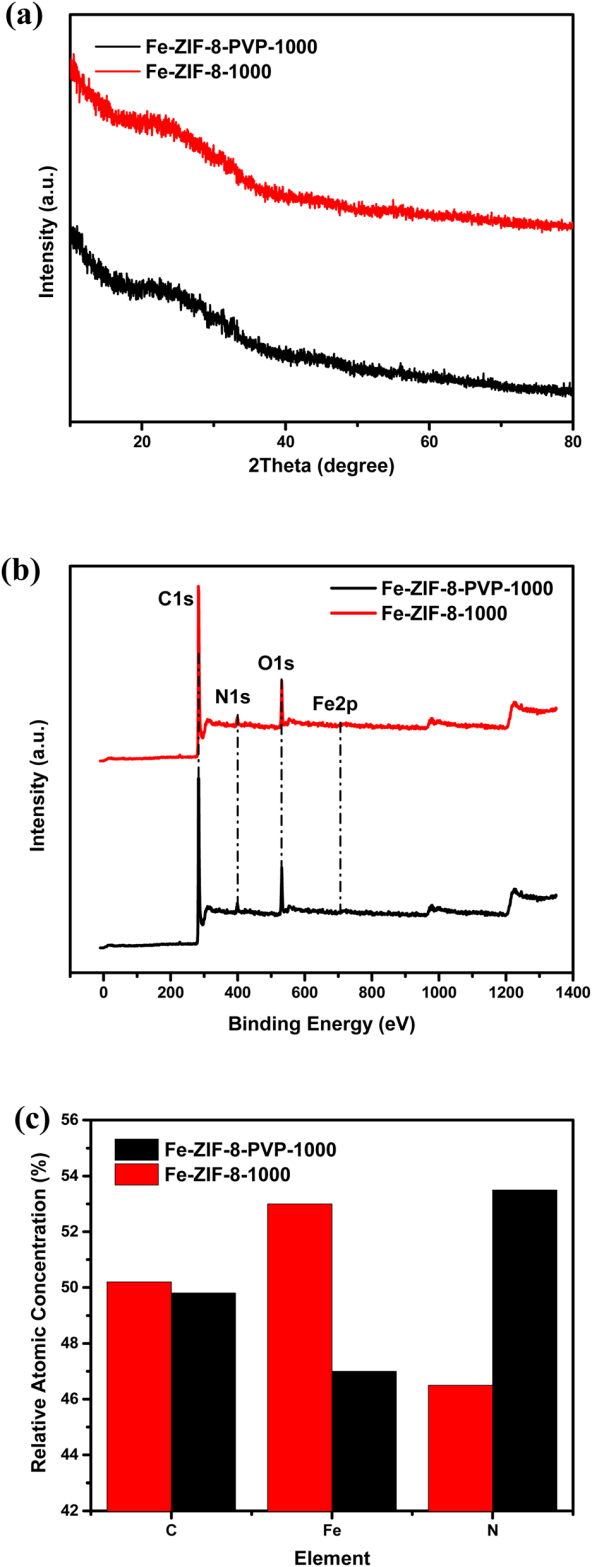
(a) XRD patterns of Fe-ZIF-8-PVP-1000 and Fe-ZIF-8-1000, (b) XPS survey spectrum of Fe-ZIF-8-PVP-1000 and Fe-ZIF-8-1000, and (c) relative atomic distribution (by XPS) in Fe-ZIF-8-1000 and Fe-ZIF-8-PVP-1000.

The X-ray diffraction (XRD) patterns of Fe-ZIF-8-1000 and Fe-ZIF-8-PVP-1000 (Fig. 3a) exhibit broad, low-intensity humps centered around 2*θ* ≈ 24–26°, which correspond to the (002) diffraction plane of turbostratic or disordered graphitic carbon.^78^ The broad nature of the peak and the absence of sharp reflections confirm that both materials possess a largely amorphous carbon structure, typical of metal–organic framework-derived carbons after high-temperature pyrolysis.^79^ Importantly, no characteristic diffraction peaks associated with crystalline iron or iron-based compounds (such as Fe, Fe_3_C, Fe_2_O_3_, or Fe_3_O_4_) were observed in either sample. This suggests that the Fe species are either atomically dispersed, embedded as ultra-small clusters below the XRD detection limit, or encapsulated within the carbon matrix—making them undetectable by conventional XRD.^79,80^Fig. 3b presents the XPS survey spectra of Fe-ZIF-8-1000 and Fe-ZIF-8-PVP-1000, showing distinct peaks corresponding to C 1s (∼284.8 eV), N 1s (∼399.8 eV), O 1s (∼531.8 eV), and a very weak Fe 2p signal (∼711–725 eV).^18,81^ The presence of these elements confirms the successful incorporation of nitrogen and a small amount of iron into the carbon framework. Fig. 3c depicts a comparison of the elemental content (%) of carbon (C), iron (Fe), and nitrogen (N) in Fe-ZIF-8-1000 and Fe-ZIF-8-PVP-1000 as determined by X-ray photoelectron spectroscopy (XPS). The Fe-ZIF-8-PVP-1000 sample exhibits considerably higher nitrogen content, and a lower iron content compared to Fe-ZIF-8-1000, with no significant difference observed in the carbon content between the two samples. The incorporation of PVP during synthesis appears to enrich nitrogen content while suppressing excessive iron incorporation. This compositional tuning indicates that PVP promotes the formation of more uniform and isolated Fe–N_*x*_ coordination structures by creating a nitrogen-rich environment and limiting iron aggregation, which is beneficial for enhancing the performance of single-atom catalysts.^82^

**Fig. 4:**
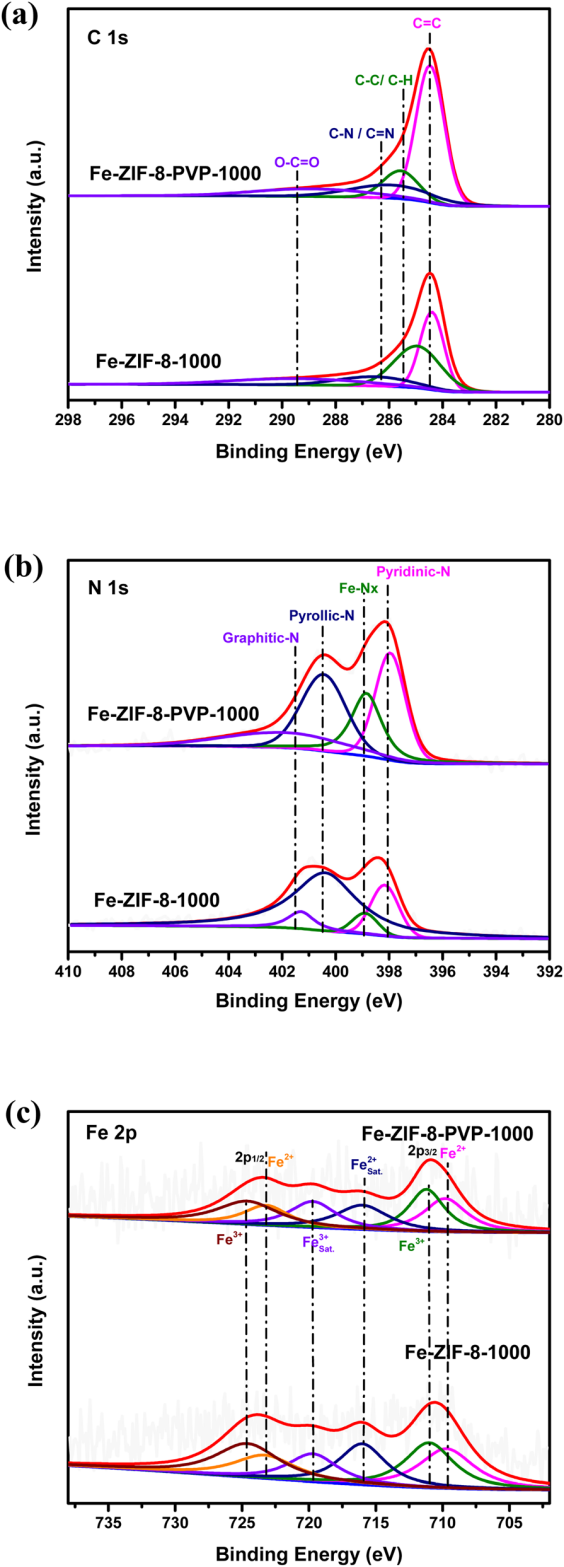
Detailed high-resolution XPS scans for Fe-ZIF-8-PVP-1000 and Fe-ZIF-8-1000: (a) C 1s peak, (b) N 1s peak, and (c) Fe 2p peak.

As illustrated in Fig. 4a, the high-resolution C 1s XPS spectra were recorded for Fe-ZIF-8-PVP-1000 and Fe-ZIF-8-1000, revealing insights into the surface carbon functionalities of the two materials. Both samples exhibit deconvoluted peaks corresponding to graphitic carbon (C=C, ∼284.6 eV), aliphatic carbon (C–C/C–H, ∼285.2 eV), nitrogen-containing groups (C–N/C=N, ∼286.3 eV), and oxygenated species (O–C=O, ∼288.7 eV).^83,84^ Compared with Fe-ZIF-8-1000, the Fe-ZIF-8-PVP-1000 sample shows a relatively stronger contribution from the graphitic carbon and C–N/C=N components, reflecting the incorporation of nitrogen-containing groups likely derived from PVP. This trend is consistent with N-doped carbons, where C–N/C=N appears at higher binding energy than C=C due to heteroatom polarization, while increased sp^2^ content enhances electronic transport across the carbon matrix and facilitates interfacial charge transfer during ORR.^83–85^ In this context, the PVP-assisted route not only promotes nitrogen functionality but also enhances graphitization, two features known to strengthen coupling between the carbon host and Fe–N_*x*_ sites.^27,86^ Importantly, Fe-ZIF-8-PVP-1000 exhibits a slight positive shift in the C–C/C–H component and a negative shift for C–N/C=N. This opposite trend reflects charge redistribution between sp^2^-hybridized carbon and nitrogen-doped sites, suggesting that PVP-assisted synthesis enhances electron delocalization across the carbon framework.^87^ Such redistribution is expected to improve conductivity and optimize charge transfer kinetics, both critical for ORR catalysis.

Fig. 4b displays the high-resolution N 1s XPS spectra obtained for Fe-ZIF-8-PVP-1000 and Fe-ZIF-8-1000. The spectra highlight the diversity of nitrogen species incorporated into the carbon skeleton, shedding light on the electronic environment and potential contributions of these sites to catalytic activity. The deconvoluted peaks correspond to pyridinic-N (∼398 eV),^88^ Fe–N_*x*_ (∼399 eV),^89^ pyrrolic-N (∼400.4 eV),^90^ and graphitic-N (∼401.5 eV),^91^ all of which are commonly found in Fe–N–C catalysts and contribute to their electrochemical activity. Compared with Fe-ZIF-8-1000, the Fe-ZIF-8-PVP-1000 sample displays higher proportions of pyridinic-N and Fe–N_*x*_ together with a slight negative binding-energy shift. Such reproducible downshifts of ≈0.05–0.15 eV reflect an electron-rich local environment, arising from stronger donation of N 2p electrons to Fe centers and the formation of more covalent Fe–N interactions.^92,93^ This redistribution of charge density has been correlated with weaker binding of oxygenated intermediates, thereby positioning the Fe–N–C system closer to the Sabatier optimum for oxygen reduction.^92,94–98^ Because pyridinic-anchored FeN_4_ moieties are widely regarded as the most active catalytic sites in acidic media, and molecular FeN_4_ analogues closely replicate their spectroscopic signatures, the observed N 1s features provide strong evidence for electron-rich Fe–N_4_ environments that are optimally configured to tune the binding strength of O_2_ and OOH* intermediates.^92^ Although Fe-ZIF-8-PVP-1000 contains lower Fe content, its higher nitrogen content and improved dispersion result in a larger fraction of Fe atoms being stabilized as Fe–N_*x*_ moieties, accounting for the stronger Fe–N_*x*_ peak observed in the N 1s spectrum compared to Fe-ZIF-8-1000. Beyond the enrichment of pyridinic-N and Fe–N_*x*_, the PVP-assisted material also exhibits a larger fraction of graphitic-N. This structural component not only strengthens the π-electron backbone conductivity but also acts synergistically with pyridinic-N to accelerate charge transport and promote favorable reaction kinetics.^92,99^ As a result, this cooperative environment contributes directly to enhanced ORR activity.^95,100^ On the other hand, pyrrolic-N is slightly more abundant in Fe-ZIF-8-1000, but it is generally considered less stable and less catalytically active under ORR conditions.^101,102^ These differences suggest that the presence of PVP during synthesis promotes the formation of catalytically favorable nitrogen species, likely by influencing the nitrogen retention and coordination environment during pyrolysis. As a result, Fe-ZIF-8-PVP-1000 is expected to demonstrate enhanced ORR performance due to its enriched population of active nitrogen functionalities.

Fig. 4c presents the high-resolution Fe 2p XPS spectra of Fe-ZIF-8-1000 and Fe-ZIF-8-PVP-1000. Both samples exhibit well-defined Fe 2p_3/2_ and Fe 2p_1/2_ peaks, along with characteristic satellite features, indicative of the presence of mixed-valence iron species. For Fe-ZIF-8-1000, the deconvoluted Fe 2p_3/2_ spectrum displays two prominent peaks at 709.7 eV and 711.0 eV, corresponding to Fe^2+^ and Fe^3+^ species, respectively, accompanied by satellite peaks at 716.0 eV and 719.7 eV, while the associated Fe 2p_1/2_ signals appear at 723.3 eV (Fe^2+^) and 724.6 eV (Fe^3+^), consistent with the characteristic spin–orbit splitting of iron.^103–105^ A comparable spectral profile was observed for Fe-ZIF-8-PVP-1000, confirming coexistence of Fe^2+^ and Fe^3+^; this reflects partial reduction of Fe^3+^ during pyrolysis and the generation of redox-active Fe centers relevant to ORR. Notably, Fe-ZIF-8-PVP-1000 exhibits a slight positive shift in Fe 2p_3/2_ and a negative shift in Fe 2p_1/2_. This asymmetric behavior points to subtle modification of the Fe electronic environment: PVP-derived nitrogen ligation increases electron donation to Fe–N_*x*_ centers (as also indicated by N 1s shifts) while altering Fe spin states and the Fe^2+^/Fe^3+^ distribution. At very low Fe loadings, however, such counter-shifts are often attributed to multiplet reweighting and final-state effects rather than genuine changes in oxidation state, making the N 1s downshifts the more reliable indicator of electron-density redistribution at Fe–N_*x*_.^105–107^ The overall Fe 2p signal intensity was lower in Fe-ZIF-8-PVP-1000 than in Fe-ZIF-8-1000, consistent with ICP-OES measurements (0.4765 wt% *vs.* 0.8813 wt%). This quantitative difference highlights the role of PVP in suppressing Fe aggregation and favoring atomically dispersed Fe sites. The weaker Fe signals and broader peak shapes further support the single-atom dispersion model, where electronic isolation of Fe centers enhances catalytic selectivity and durability.^108,109^

**Fig. 5:**
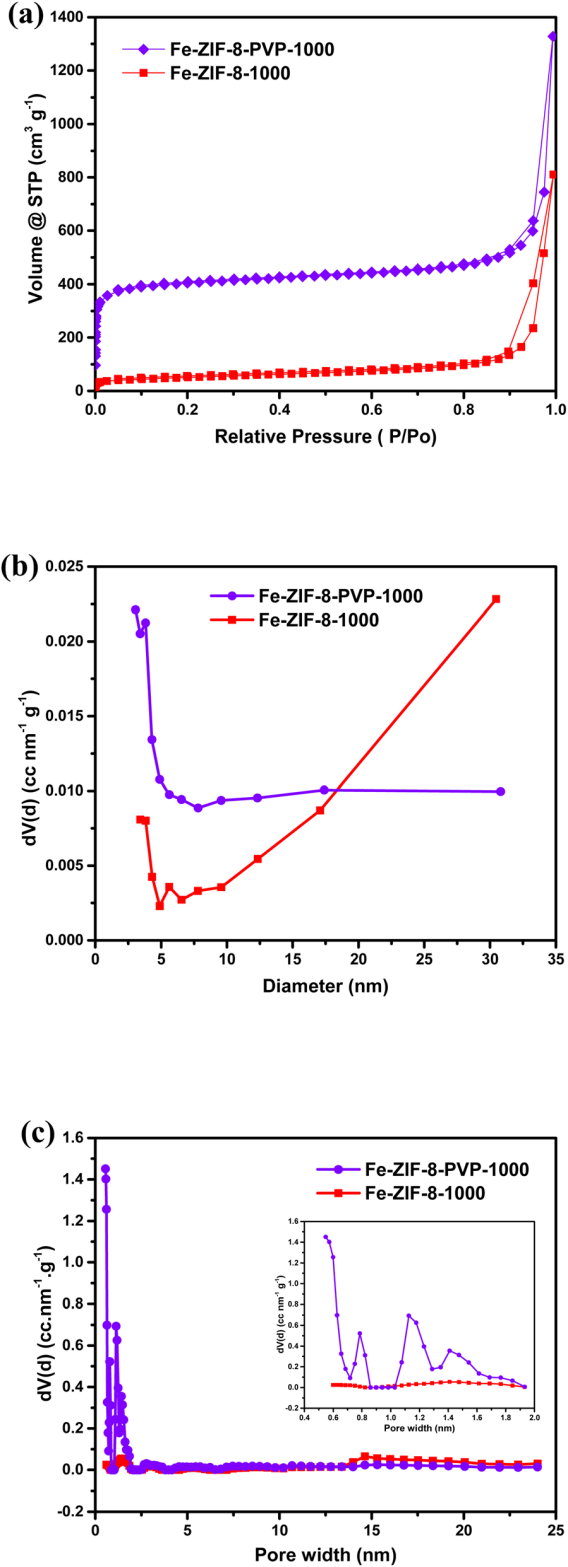
(a) N_2_ adsorption curves of Fe-ZIF-8-PVP-1000 and Fe-ZIF-8-1000; (b) BJH-derived mesoporous size distribution patterns of Fe-ZIF-8-PVP-1000 and Fe-ZIF-8-1000; (c) DFT pore size distribution of Fe-ZIF-8-1000 and Fe-ZIF-8-PVP-1000 with emphasis on micropore region.

Fig. 5a shows distinct isotherm characteristics for the two samples. The Fe-ZIF-8-PVP-1000 sample shows a rapid rise in nitrogen uptake in the low-pressure region (*P*/*P*_0_ < 0.1), consistent with a Type I isotherm—a signature of microporous frameworks.^110^ In contrast, Fe-ZIF-8-1000 displays a broader uptake over an extended *P*/*P*_0_ range with a discernible hysteresis loop, indicative of Type IV behavior associated with mesoporous or disordered carbon structures.^111^ This divergence features the role of PVP as a structure-directing agent during pyrolysis, effectively preserving the microporous network. The t-plot method based on the DeBoer thickness model further confirmed the enhanced microporosity in Fe-ZIF-8-PVP-1000, with a micropore volume of 0.540 cm^3^ g^−1^ and a micropore surface area of 645.5 m^2^ g^−1^, while the non-PVP counterpart had significantly lower values: 0.035 cm^3^ g^−1^ and 7.9 m^2^ g^−1^, respectively, as can be seen in Table S1. This substantial difference highlights the effectiveness of PVP in preserving micropores, which are essential for the anchoring and accessibility of single-atom Fe sites. Results from the Dubinin–Radushkevich (DR) method corroborated the *t*-plot findings. The Fe-ZIF-8-PVP-1000 sample exhibited a micropore volume of 0.642 cm^3^ g^−1^, an average pore width of 1.44 nm, and an adsorption energy of 18.0 kJ mol^−1^—values indicative of a uniform and energetically favorable microporous environment. Conversely, Fe-ZIF-8-1000 showed a wider average pore width (2.15 nm), lower micropore volume (0.077 cm^3^ g^−1^), and lower adsorption energy (12.1 kJ mol^−1^), again reflecting pore coarsening and potential framework collapse during carbonization.

The specific surface area of Fe-ZIF-8-PVP-1000, determined by the BET method was remarkably high, reaching 1579.8 m^2^ g^−1^, compared to 177.3 m^2^ g^−1^ for Fe-ZIF-8-1000. The corresponding BET C constants (879.6 *vs.* 232.7) and high linear correlation coefficients (*r* ≈ 1.000) reflect strong adsorbate–adsorbent interactions and excellent data reliability. Further insight into mesoporosity was obtained using the BJH desorption model as shown in Fig. 5b. The Fe-ZIF-8-PVP-1000 sample exhibited a narrow mesopore size distribution centered at ∼3.8 nm, with a total BJH pore volume of 0.382 cm^3^ g^−1^ and mesopore surface area of 109.7 m^2^ g^−1^. In contrast, Fe-ZIF-8-1000 demonstrated a broader distribution peaking around 30.5 nm, with a higher total pore volume of 0.573 cm^3^ g^−1^ and mesopore surface area of 100.5 m^2^ g^−1^. Pore structure precision was refined using Density Functional Theory (DFT) analysis applied to the adsorption branch, which is presented in Fig. 5c. The PVP-assisted material exhibited a dominant micropore mode at 0.548 nm, with a surface area of 1747.3 m^2^ g^−1^ and a pore volume of 0.916 cm^3^ g^−1^. In contrast, Fe-ZIF-8-1000 showed a broader pore width mode at 14.6 nm, and significantly reduced surface area and pore volume (148.2 m^2^ g^−1^ and 0.563 cm^3^ g^−1^, respectively). This shift reflects the transformation from a microporous to a mesoporous framework in the absence of PVP, consistent with DFT's sensitivity to subtle pore geometry changes. These observations confirm that Fe-ZIF-8-PVP-1000 possesses a hierarchical porous architecture, marked by significantly enhanced microporosity and a sufficient level of mesoporosity. The micropores facilitate high surface area and Fe–N_*x*_ site dispersion, while the mesopores improve ion diffusion and mass transport. In contrast, Fe-ZIF-8-1000 demonstrates an almost entirely mesoporous architecture, indicating minimal residual microporosity.

**Fig. 6:**
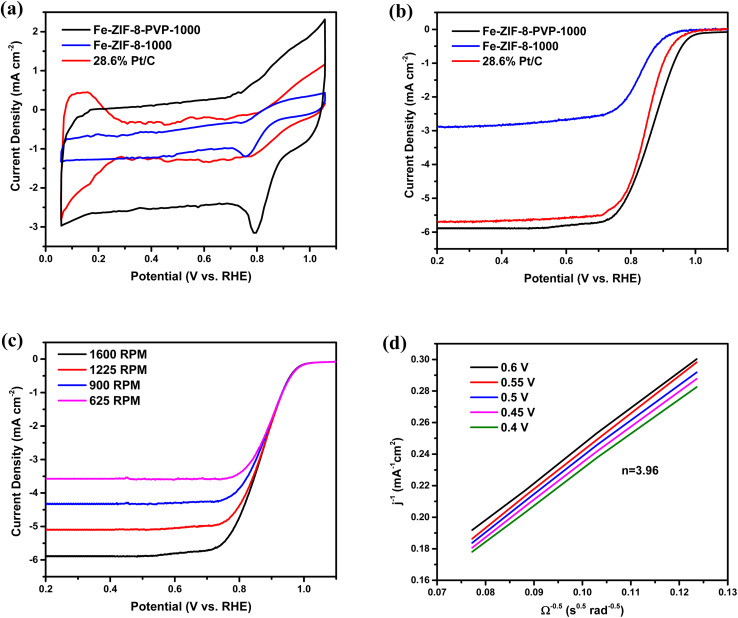
Electrochemical ORR performance: (a) cyclic voltammetry curves of Fe-ZIF-8-PVP-1000, Fe-ZIF-8-1000, and commercial 28.6% Pt/C catalysts recorded in O_2_-saturated 0.5 M H_2_SO_4_ at a sweep rate of 10 mV s^−1^ and a rotation speed of 1600 rpm, (b) linear sweep voltammetry profiles of Fe-ZIF-8-PVP-1000, Fe-ZIF-8-1000, and 28.6% Pt/C catalysts measured in O_2_-saturated 0.5 M H_2_SO_4_ at a rotation speed of 1600 rpm, (c) LSV profiles of Fe-ZIF-8-PVP-1000 in O_2_-saturated 0.5 M H_2_SO_4_ at a scan rate of 10 mV s^−1^ at different RDE rotation rates, (d) K–L plots of Fe-ZIF-8-PVP-1000 at different potentials.

The electrocatalytic performance of the Fe-ZIF-8-PVP-1000 catalyst toward the oxygen reduction reaction (ORR) was systematically investigated using a suite of electrochemical techniques. Fig. 6a presents the cyclic voltammograms (CVs) of Fe-ZIF-8-1000, Fe-ZIF-8-PVP-1000, and commercial 28.6% Pt/C, recorded in O_2_-saturated 0.5 M H_2_SO_4_. All measurements were conducted under identical conditions to allow a direct comparison of their ORR activities. The Fe-ZIF-8-1000 catalyst exhibits reduced ORR activity, as evidenced by a significantly lower onset potential and cathodic current, which suggests minimal ORR kinetics, likely due to limited active site accessibility and poor electrical conductivity.^112^ Remarkably, the incorporation of PVP during Fe-ZIF-8 synthesis leads to a dramatic enhancement in ORR activity. The Fe-ZIF-8-PVP-1000 sample exhibits a more positive onset potential compared to both Fe-ZIF-8-1000 and commercial Pt/C. Strikingly, the PVP-modified catalyst achieves a cathodic current density approximately three times higher than both the non-PVP sample and Pt/C, highlighting its superior ORR kinetics and active site utilization. This remarkable performance is attributed to the PVP-induced homogeneous dispersion of Fe–N_*x*_ active sites, as well as improved electronic conductivity and interaction within the carbon matrix.^60,87,113^ The state-of-the-art commercial Pt/C (28.6%) catalyst, used here as a practical benchmark, displays the expected well-defined cathodic wave indicative of high ORR activity, with a half-wave potential (*E*_1/2_) slightly lower than that of Fe-ZIF-8-PVP-1000. Interestingly, the Fe-ZIF-8-PVP-1000 catalyst delivers a noticeably higher current density, underscoring its potential to surpass commercial Pt/C catalyst.

Fig. 6b presents the linear sweep voltammetry (LSV) curves recorded under O_2_-saturated conditions, offering further insight into the electrocatalytic activity. The Fe-ZIF-8-PVP-1000 catalyst demonstrates an outstanding half-wave potential (*E*_1/2_) of 0.865 V, which is among the highest reported for advanced Fe–N–C-based ORR catalysts in acidic media, as summarized in Table S2. Remarkably, its *E*_1/2_ value even exceeds that of the commercial 28.6% Pt/C catalyst (0.855 V), long regarded as the benchmark for ORR activity, highlighting its exceptional promise as a platinum-group-metal-free alternative. The high *E*_1/2_ value reflects favorable reaction kinetics and low overpotential for the subsequent electron-transfer steps.^114^ In contrast, Fe-ZIF-8-1000, synthesized without PVP, displays a significantly lower half-wave potential of 0.82 V, underscoring the critical role of PVP in optimizing active site distribution and electronic conductivity. To probe the ORR mechanism and quantify the kinetics of the Fe-ZIF-8-PVP-1000 catalyst, LSVs were recorded at varying rotation speeds using a rotating disk electrode (RDE). As shown in Fig. 6c, the diffusion-limited current increases with rotation rate, reflecting improved O_2_ mass transport at the catalyst–electrolyte interface. The corresponding Koutecky–Levich (K–L) plots (Fig. 6d), constructed from the LSV data, exhibit good linearity across the tested potentials, indicating first-order reaction kinetics with respect to dissolved oxygen. The nearly parallel slopes at different potentials suggest a consistent and well-defined reaction pathway.^115^ Based on the K–L analysis, the calculated electron transfer number (*n* = 3.96) is very close to 4.0, signifying a predominant four-electron reduction pathway. This implies the direct reduction of O_2_ to H_2_O without the formation of significant quantities of H_2_O_2_, a crucial characteristic for high-efficiency ORR catalysts.^116^ The observed four-electron pathway is attributed to the atomically dispersed Fe centers coordinated to nitrogen species within a conductive carbon matrix, which facilitates effective O–O bond cleavage and minimizes peroxide formation.

**Fig. 7:**
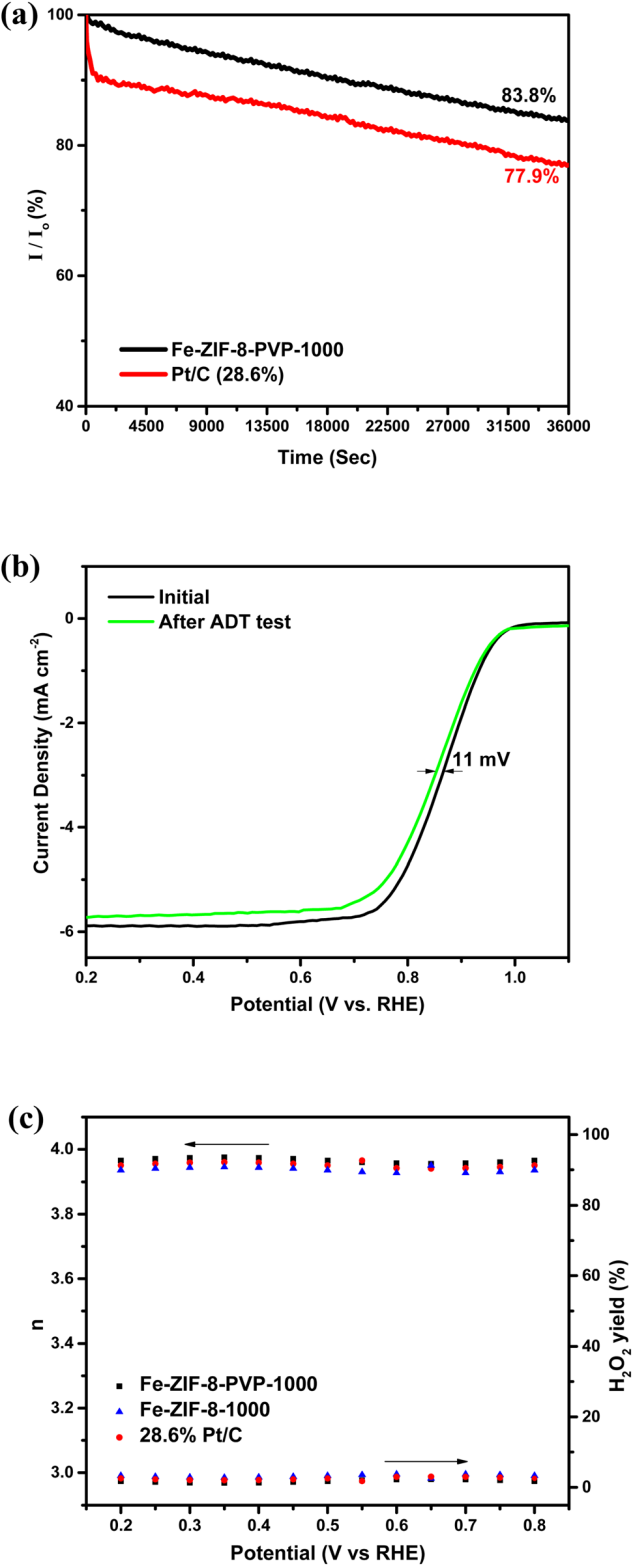
(a) Chronoamperometric response of Fe-ZIF-8-PVP-1000 and 28.6% Pt/C catalysts in O_2_-saturated 0.5 M H_2_SO_4_ at 0.8 V *versus* RHE. (b) LSVs of the catalysts before and after 30 000-cycle ADT in O_2_-saturated 0.5 M H_2_SO_4_ at 1600 rpm and 10 mV s^−1^. (c) H_2_O_2_ yield and calculated electron transfer number of Fe-ZIF-8-PVP-1000, Fe-ZIF-8-1000, and Pt/C in O_2_-saturated 0.5 M H_2_SO_4_ at 1600 rpm.

Long-term operational stability is essential for the practical deployment of ORR catalysts in energy devices. The stability of the catalyst was examined through chronoamperometric measurements in 0.5 M H_2_SO_4_ with continuous oxygen bubbling. The test was conducted at a rotation speed of 1600 rpm, while maintaining the potential at 0.8 V *versus* RHE. As shown in Fig. 7a, Fe-ZIF-8-PVP-1000 maintained approximately 84% of its initial current density after 10 h of potentiostatic operation, clearly outperforming commercial Pt/C under the same conditions. Furthermore, the durability of the catalyst was evaluated in 0.5 M H_2_SO_4_ through continuous potential cycling between 0.6 and 1.0 V *versus* RHE at a scan rate of 50 mV s^−1^.^69–71^ As shown in Fig. 7b, Fe-ZIF-8-PVP-1000 demonstrated remarkable stability under the accelerated durability test (ADT) conditions in acidic medium, with only an 11 mV negative shift in *E*_1/2_ after 30 000 cycles. This superior stability and durability are ascribed to the robust carbon framework formed during pyrolysis and the strong coordination environment of Fe–N_*x*_ active centers, which are resistant to dissolution and agglomeration in acidic environments.

To further probe the reaction pathway, RRDE analysis was performed on both Fe-ZIF-8-PVP-1000 and the Pt/C benchmark (Fig. 7c). The Fe-ZIF-8-PVP-1000 catalyst delivered an average electron transfer number of 3.97 over the potential range of 0.2–0.8 V,^117,118^ marginally higher than that of Pt/C (3.95), confirming its near-complete four-electron reduction pathway. Consistently, the average H_2_O_2_ yield was as low as 1.75%, compared with 2.42% for Pt/C, underscoring the superior selectivity of Fe-ZIF-8-PVP-1000 toward the desired four-electron ORR process. These results demonstrate that Fe-ZIF-8-PVP-1000 combines favorable kinetics with excellent selectivity, highlighting its strong potential as an efficient ORR electrocatalyst.

## 4 Conclusion

This study presents a rationally engineered Fe–N–C catalyst derived from a polyvinylpyrrolidone (PVP)-modified ZIF-8 precursor, namely Fe-ZIF-8-PVP-1000, that achieves remarkable oxygen reduction reaction (ORR) activity and durability in acidic media. By strategically integrating PVP during synthesis, we realize a notable breakthrough in tackling longstanding challenges in Fe–N–C catalyst development: Fe aggregation, micropore collapse, and the formation of suboptimal nitrogen coordination environments. Advanced structural and spectroscopic analyses reveal that PVP acts synergistically as a morphological stabilizer, nitrogen dopant, and metal-dispersing agent, yielding atomically dispersed Fe–N_*x*_ sites embedded within a hierarchically porous carbon matrix. Compared to its PVP-free analogue, Fe-ZIF-8-PVP-1000 exhibits a markedly enhanced micropore volume, higher nitrogen content (particularly catalytically active pyridinic-N and Fe–N_*x*_ species), and a drastically increased BET surface area—all of which contribute to optimal active site exposure and electronic conductivity.

Electrochemical evaluation establishes Fe-ZIF-8-PVP-1000 as a platinum-free ORR catalyst with benchmark-beating performance. It achieves a half-wave potential (*E*_1/2_) of 0.865 V *vs.* RHE in 0.5 M H_2_SO_4_, outperforming commercial 28.6% Pt/C (*E*_1/2_ ≈ 0.855 V) under identical conditions, and exhibits nearly threefold higher cathodic current density compared to both the non-PVP analogue and Pt/C. Furthermore, it follows a near-ideal four-electron ORR pathway (*n* ≈ 3.96) and maintains 84% of its initial current after 10 hours of continuous operation, highlighting its outstanding durability in acidic environments—a critical weakness for most NPMCs. Notably, under accelerated durability test (ADT) conditions, Fe-ZIF-8-PVP-1000 exhibits only an 11 mV negative shift in *E*_1/2_ after 30 000 cycles, underscoring its superior long-term stability. In addition, RRDE measurements confirm a near-complete four-electron pathway with minimal H_2_O_2_ yield, further validating its high selectivity and efficiency. Beyond the empirical achievements, this work offers mechanistic insight into the role of PVP in modulating carbon microstructure, nitrogen functionality, and Fe coordination, shedding light on the molecular-level phenomena governing active site evolution during MOF pyrolysis. The results validate the hypothesis that PVP not only preserves microporosity and enhances Fe dispersion but also selectively fosters the formation of catalytically superior nitrogen species. These findings mark a significant advancement in the rational design of acid-tolerant NPMCs. The insights garnered herein lay the groundwork for next-generation Fe–N–C materials with engineered porosity, optimized coordination chemistry, and durable activity in harsh electrochemical environments—key attributes required for high-performance, long-term durable fuel cell technologies.

## Conflicts of interest

There are no conflicts to declare.

## Supplementary Material

RA-015-D5RA05653E-s001

## Data Availability

Additional datasets are available from the corresponding author upon reasonable request. All data supporting the results and conclusions of this study are included within this article and its SI. See DOI: https://doi.org/10.1039/d5ra05653e.
