# Supplementary material for: Fe–N–C catalyst with atomic Fe dispersion and hierarchical porosity via PVP-assisted MOF synthesis for ORR in acidic media
Source: RSC Adv. 2025 Sep 22;15(41):34524–36. doi: 10.1039/d5ra05653e (PMC12451445; doi:10.1039/d5ra05653e)
Supplement: RA-015-D5RA05653E-s001 [file RA-015-D5RA05653E-s001.pdf]

Supplementary Information (SI) for RSC Advances.
This journal is © The Royal Society of Chemistry 2025

SUPPLEMENTARY INFORMATION

Fe–N–C Catalyst with Atomic Fe Dispersion and Hierarchical Porosity via PVP-Assisted MOF Synthesis for ORR in Acidic Media

Muhammad Shoaib,[a] Shagufta Ishtiaque,[a] Ahsan Abdul Ghani,[*a] Awan Zahoor,[b] Abid Ali,[c] Ayisha Rubab[a] and Maaz Akhtar[d]
[a]Department of Chemical Engineering, University of Karachi, Karachi, Pakistan
[b]Department of Food Engineering, NED University of Engineering and Technology, Karachi, Pakistan
[c]Chongqing Key Laboratory for Advanced Materials and Technologies of Clean Energies, School of Materials and Energy, Southwest University, Chongqing 400715, PR China
[d]Industrial Engineering Department, College of Engineering, Imam Mohammad Ibn Saud Islamic University (IMSIU), Riyadh, KSA

Experimental Section

Materials

2-Methylimidazole (MeIm, $C_4H_6N_2$, 99%), polyvinylpyrrolidone (PVP, M.W. ~10,000), zinc nitrate hexahydrate ($Zn(NO_3)_2 \cdot 6H_2O$, 99%), and iron(III) nitrate nonahydrate ($Fe(NO_3)_3 \cdot 9H_2O$, 98%) were all purchased from Sigma-Aldrich. Nafion solution (5 wt%) and sulfuric acid (H_2SO_4, 98%) were obtained from Alfa Aesar. Methanol and ethanol were of analytical grade. All reagents were used as received without further purification, and ultrapure water (18.2 MΩ·cm) was used in all procedures where applicable.

Structural and Morphological Characterization

X-ray diffraction (XRD) patterns were collected using a PANalytical X'Pert PRO diffractometer equipped with Cu Kα radiation (λ = 1.5406 Å) to analyze the crystallographic structure of the catalysts. Scans were performed over a 2θ range of 10° to 80° at a rate of 4° min^{-1} with a 0.2 s step time. Surface morphology was examined using field-emission scanning electron microscopy (FESEM, Zeiss Ultra55) operating at an accelerating voltage of 5 kV. Transmission electron microscopy (TEM) imaging was performed on a Hitachi H-9500 microscope at an accelerating voltage of 300 kV to probe internal microstructures and particle dispersity.

Textural and Surface Area Analysis

Nitrogen adsorption–desorption isotherms were measured at 77 K using a Quantachrome Autosorb-1-C surface area analyzer. Prior to measurements, all samples were degassed under vacuum (~10^{-6} Pa) at 200 °C for 2 h. The Brunauer–Emmett–Teller (BET) method was used to determine the specific surface area, while micropore volumes and areas were estimated using t-plot and Dubinin–Radushkevich (DR) models. Pore size distributions were derived using both the Barrett–Joyner–Halenda (BJH) and density functional theory (DFT) models applied to the adsorption branches of the isotherms.

X-ray Photoelectron Spectroscopy (XPS)

XPS analysis was conducted using a PHI-5000C ESCA system (PerkinElmer) with Mg Kα radiation (hν = 1253.6 eV) to evaluate surface elemental composition and chemical states. The spectra were processed, fitted, and deconvoluted using XPS Peak 4.1 software to distinguish individual bonding environments.

Elemental Analysis (ICP-OES)

Quantitative determination of iron content was performed using an Agilent 5900 inductively coupled plasma optical emission spectrometer (ICP-OES). For digestion, samples were first calcined at 650 °C for 1 h in a muffle furnace. The resulting ash was transferred to a PTFE crucible, treated with 8 mL HNO_3 and 3 mL HCl, and digested on a hotplate to near dryness. The digested solution was diluted to a final volume of 25 mL with deionized water prior to analysis.

Electrochemical Measurements

The electrocatalytic activity of the synthesized catalysts toward the oxygen reduction reaction (ORR) was investigated using a CHI 1140A electrochemical workstation (CH Instruments) in a standard three-electrode cell configuration. Catalyst inks were prepared by dispersing 8.0 mg of catalyst in 3 mL ethanol and 0.2 mL of 5 wt% Nafion, followed by ultrasonication. A glassy carbon electrode (3 mm diameter) served as the working electrode, onto which 20 µL of catalyst ink was drop-cast and air-dried. A saturated calomel electrode (SCE) and Pt wire in saturated KCl solution were used as reference and counter electrodes**, respectively, and connected via a salt bridge. Cyclic voltammetry (CV) was conducted in O_2-saturated 0.5 M H_2SO_4 at 25 °C, with a scan rate of 10 mV s^{-1}. All potentials were referenced to the reversible hydrogen electrode (RHE) using the equation:

E(V vs. RHE) = E(V vs. SCE) + 0.241 + 0.0591 x pH, at 25 C

Linear sweep voltammetry (LSV) was performed using a rotating disk electrode (RDE-2, BASi Inc.) and the electron transfer number (n) for the oxygen reduction reaction (ORR) was estimated using the Koutecký–Levich equation as follows:

$$1/j = 1/jk + 1/jd = 1/jk + 1 / (0.62\ n\ F\ D_O^{2/3}\ v^{-1/6}\ C_O\ \omega^{1/2})$$

In this model, F is the Faraday constant (96,485 C mol^{-1}) while j, jd, and jk represent the overall measured current density, the diffusion-limited current density, and the kinetic current density, respectively. For experiments conducted in 0.5 M H_2SO_4, key parameters specific to the acidic medium were used: the oxygen diffusion coefficient (D_O) is taken as 1.4×10^{-5} $cm^2\ s^{-1}$, the solubility of oxygen (C_O) is 1.26×10^{-6} mol cm^{-3}, and the kinematic viscosity (ν) is 0.01 $cm^2\ s^{-1}$. The electrode rotation speed (ω) was varied to generate Koutecký–Levich (K–L) plots, which relate j^{-1} and $\omega^{-1/2}$. These plots were obtained from linear sweep voltammograms recorded at different rotation rates. The slope of each K–L line was then used to calculate the number of electrons involved in the ORR, providing insights into the underlying reaction mechanism in acidic conditions.

RRDE measurements were used to calculate the electron transfer number (*n*) and H_2O_2 yield (%H_2O_2) using following equations:

$$H_2O_2\% = 200 \times \frac{I_r/N}{I_d + I_r/N}$$

$$n = 4 \times \frac{I_d}{I_d + I_r/N}$$

where I_d is the disk current, I_r is the ring current, and N is the collection efficiency (0.37) of the ring electrode. The geometric area of the RRDE was 0.2475 cm^2 for the disk and 0.188 cm^2 for the ring.

** *Note: A platinum wire was used as the counter electrode during electrochemical measurements. This choice is consistent with widely reported ORR studies on Fe–N–C catalysts [Tan et al., Surf. Interfaces 56, 105683 (2025); Liu et al., J. Alloys Compd. 1036, 182115 (2025); Wang et al., Angew. Chem. Int. Ed. 64, e202510671 (2025); Cui et al., Ind. Chem. Mater. 2, 634–643 (2024)]. Although*

Pt dissolution and redeposition have been discussed in the literature, such effects are negligible under the applied conditions. Therefore, the use of a Pt counter electrode does not affect the intrinsic activity evaluation of Fe-ZIF-8-PVP-1000.

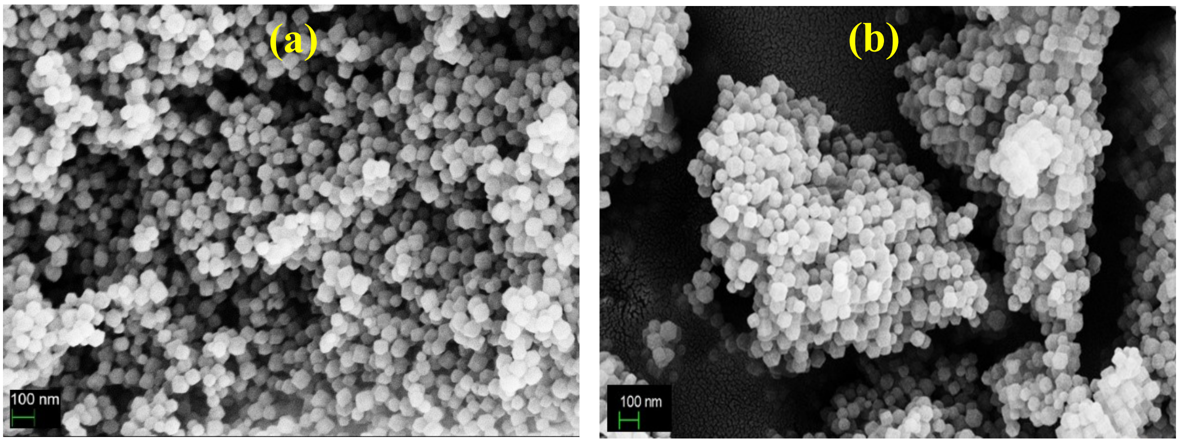

Figure S1. SEM images of (a) Fe-ZIF-8-PVP and (b) Fe-ZIF-8.

Table S1: Pore characteristics of the catalysts

Parameter	Fe-ZIF-8-PVP-1000	Fe-ZIF-8-1000
BET Surface Area (m^2/g)	1579.8	177.3
BJH Pore Volume (cm^3/g)	0.382	0.573
BJH Pore Diameter (nm)	3.816	30.47
BJH Mesopore Surface Area (m^2/g)	109.7	100.5
Micropore Volume (t-Plot) (cm^3/g)	0.54	0.035
Micropore Surface Area (t-Plot) (m^2/g)	645.5	7.9
Micropore Volume (DR) (cm^3/g)	0.642	0.077
Average Pore Width (DR) (nm)	1.44	2.15
Adsorption Energy (DR) (kJ/mol)	18.015	12.096
Total Pore Volume (DFT) (cm^3/g)	0.916	0.563
Total Surface Area (DFT) (m^2/g)	1747.3	148.2
Pore Width Mode (DFT) (nm)	0.548	14.636

Table S2. Comparison of ORR half-wave potentials in acid media between this study and previously reported iron-based catalysts.

Catalyst	Electrolyte	$E_{1/2}$ vs RHE	Reference
Fe-ZIF-8-PVP-1000	0.5 M H_2SO_4	0.865 V	This work
FePNC	0.5 M H_2SO_4	0.76 V	(1)
FeSNC	0.5 M H_2SO_4	0.76 V	(2)
RN350-Z(1-2)-1000	0.1 M $HClO_4$	0.723 V	(3)
Fe-KJB-3-60A	0.1 M $HClO_4$	0.79 V	(4)
Fe-Zn-SA/NC	0.1 M $HClO_4$	0.78 V	(5)
4.2-FeSA	0.5 M H_2SO_4	0.74 V	(6)
Fe-NCs	0.5 M H_2SO_4	0.83 V	(7)
Fe-SA/PNC	0.5 M H_2SO_4	0.86 V	(8)
$FeMn_{ac}$/Mn-N_4C	0.5 M H_2SO_4	0.79 V	(9)
Fe SAs-Fe_2P NPs/NPCFs-2.5	0.5 M H_2SO_4	0.78 V	(10)
Fe-P-NC	0.1 M $HClO_4$	0.781 V	(11)
Fe-AC-CVD	0.5 M H_2SO_4	0.846 V	(12)
FeSA/FeAC−2DNPC	0.5 M H_2SO_4	0.81 V	(13)
OM-Fe-N-C-steam	0.1 M $HClO_4$	0.81 V	(14)
FeNC-CVD−750	0.5 M H_2SO_4	0.85 V	(15)
Fe SAC-MOF−5	0.5 M H_2SO_4	0.83 V	(16)

Fe-N-C	0.5 M H_2SO_4	0.82 V	(17)
FeN4-C	0.5 M H_2SO_4	0.82 V	(18)
Fe@Fe-N-C	0.1 M $HClO_4$	0.829 V	(19)
Fe-N-C_g	0.5 M H_2SO_4	0.85 V	(20)
Fe SAs-HP	0.1 M $HClO_4$	0.78 V	(21)
FeN_5-NHC	0.1 M $HClO_4$	0.85 V	(22)
Fe-N/Cl-C/HC	0.1 M $HClO_4$	0.81 V	(23)
Fe/FeN_x-NC-1	0.5 M H_2SO_4	0.8 V	(24)
OM-Fe-NC(200)	0.5 M H_2SO_4	0.885 V	(25)
Fe_{SA}-NCc	0.5 M H_2SO_4	0.836 V	(26)
Fe(Cd)–CN_x	0.1 M $HClO_4$	0.831 V	(27)
OP-Fe-NC	0.5 M H_2SO_4	0.89 V	(28)
Cyan-Fe–N-C	0.5 M H_2SO_4	0.836 V	(29)

References

1. H. Liu, L. Jiang, Y. Sun, J. Khan, B. Feng, J. Xiao, H. Zhang, H. Xie, L. Li, S. Wang, L. Han, Asymmetric N, P-coordinated single-atomic Fe sites with Fe2P nanoclusters/nanoparticles on porous carbon nanosheets for highly efficient oxygen electroreduction, Adv. Energy Mater., 2023, 13, 2301223.

2. H. Liu, L. Jiang, Y. Sun, J. Khan, B. Feng, J. Xiao, H. Zhang, H. Xie, L. Li, S. Wang, L. Han, Revisiting the role of sulfur functionality in regulating the electron distribution of single-atomic Fe sites toward enhanced oxygen reduction, Adv. Funct. Mater., 2023, 33, 2304074.

3. M. Hao, R. Dun, Y. Su, L. He, F. Ning, X. Zhou, W. Li, In situ self-doped biomass-derived porous carbon as an excellent oxygen reduction electrocatalyst for fuel cells and metal–air batteries, J. Mater. Chem. A, 2021, 9, 14331–14343.

4. M. Wang, B. Huang, N. Jiang, T. Liu, J. Huang, L. Guan, An Fe–N–C electrocatalyst with dense active sites synthesized by expeditious pyrolysis of a natural Fe–N4 macrocyclic complex, J. Mater. Chem. A, 2022, 10, 23001–23007.

5. J. Xu, S. Lai, D. Qi, M. Hu, X. Peng, Y. Liu, W. Liu, G. Hu, H. Xu, F. Li, C. Li, J. He, L. Zhuo, J. Sun, Y. Qiu, S. Zhang, J. Luo, X. Liu, Atomic Fe–Zn dual-metal sites for high-efficiency pH-universal oxygen reduction catalysis, Nano Res., 2021, 14, 1374–1381.

6. X. Wu, Q. Wang, S. Yang, J. Zhang, Y. Cheng, H. Tang, L. Ma, X. Min, C. Tang, S. P. Jiang, F. Wu, Y. Lei, S. Ciampi, S. Wang, L. Dai, Sublayer-enhanced atomic sites of single atom catalysts through in situ atomization of metal oxide nanoparticles, Energy Environ. Sci., 2022, 15, 1183–1191.

7. S. Y. Yi, E. Choi, H. Y. Jang, S. Lee, J. Park, D. Choi, Y. Jang, H. Kang, S. Back, S. Jang, J. Lee, Insight into defect engineering of atomically dispersed iron electrocatalysts for high-performance proton exchange membrane fuel cell, Adv. Mater., 2023, 35, 2302666.

8. W. Xue, Q. Zhou, X. Cui, J. Zhang, S. Zuo, F. Mo, J. Jiang, X. Zhu, Z. Lin, Atomically dispersed FeN2P2 motif with high activity and stability for oxygen reduction reaction over the entire pH range, Angew. Chem. Int. Ed., 2023, 62, e202307504.

9. H. Liu, L. Jiang, J. Khan, X. Wang, J. Xiao, H. Zhang, H. Xie, L. Li, S. Wang, L. Han, Decorating single-atomic Mn sites with FeMn clusters to boost oxygen reduction reaction, Angew. Chem. Int. Ed., 2023, 135, e202214988.

10. Y. Pan, X. Ma, M. Wang, X. Yang, S. Liu, H. Chen, Z. Zhuang, Y. Zhang, W. Cheong, C. Zhang, X. Cao, R. Shen, Q. Xu, W. Zhu, Y. Liu, X. Wang, X. Zhang, W. Yan, J. Li, H. Chen, C. Chen, Y. Li, Construction of N, P co-doped carbon frames anchored with Fe single atoms and Fe2P nanoparticles as a robust coupling catalyst for electrocatalytic oxygen reduction, Adv. Mater., 2022, 34, 2203621.

11. H. Jin, R. Yu, P. Ji, W. Zeng, Z. Li, D. He, S. Mu, Sharply expanding single-atomically dispersed Fe–N active sites through bidirectional coordination for oxygen reduction, Chem. Sci., 2024, 15, 7259–7268.

12. S. Liu, C. Li, M. J. Zachman, Y. Zeng, H. Yu, B. Li, M. Wang, J. Braaten, J. Liu, H. M. Meyer, M. Lucero, A. J. Kropf, E. E. Alp, Q. Gong, Q. Shi, Z. Feng, H. Xu, G. Wang, D. J. Myers, J. Xie, D. A. Cullen, S. Litster, G. Wu, Atomically dispersed iron sites with a nitrogen–carbon coating as highly active and durable oxygen reduction catalysts for fuel cells, Nat. Energy, 2022, 7, 652–663.

13. X. Wan, Q. Liu, J. Liu, S. Liu, X. Liu, L. Zheng, J. Shang, R. Yu, J. Shui, Iron atom–cluster interactions increase activity and improve durability in Fe–N–C fuel cells, Nat. Commun., 2022, 13, 2963.

14. J. Zou, C. Chen, Y. Chen, Y. Zhu, Q. Cheng, L. Zou, Z. Zou, H. Yang, Facile steam-etching approach to increase the active site density of an ordered porous Fe–N–C catalyst to boost oxygen reduction reaction, ACS Catal., 2022, 12, 4517–4525.

15. L. Jiao, J. Li, L. L. Richard, Q. Sun, T. Stracensky, E. Liu, M. T. Sougrati, Z. Zhao, F. Yang, S. Zhong, H. Xu, S. Mukerjee, Y. Huang, D. A. Cullen, J. H. Park, M. Ferrandon, D. J. Myers, F. Jaouen, Q. Jia, Chemical vapour deposition of Fe–N–C oxygen reduction catalysts with full utilization of dense Fe–N4 sites, Nat. Mater., 2021, 20, 1385–1391.

16. J. Yan, H. Li, K. Wang, Q. Jin, C. Lai, R. Wang, S. Cao, J. Han, Z. Zhang, J. Su, K. Jiang, Ultrahigh phosphorus doping of carbon for high-rate sodium ion batteries anode, Adv. Energy Mater., 2021, 11, 2003911.

17. F. Xiao, X. Liu, C.-J. Sun, I. Hwang, Q. Wang, Z. Xu, Y. Wang, S. Zhu, H.-W. Wu, Z. Wei, L. Zheng, D. Cheng, M. Gu, G.-L. Xu, K. Amine, M. Shao, Solid-state synthesis of highly dispersed nitrogen-coordinated single iron atom electrocatalysts for proton exchange membrane fuel cells, Nano Lett., 2021, 21, 3633–3639.

18. G. Chen, R. Lu, C. Li, J. Yu, X. Li, L. Ni, Q. Zhang, G. Zhu, S. Liu, J. Zhang, U. I. Kramm, Y. Zhao, G. Wu, J. Xie, X. Feng, Hierarchically porous carbons with highly curved surfaces for hosting single metal FeN4 sites as outstanding oxygen reduction catalysts, Adv. Mater., 2023, 35, 2300907.

19. Z. Lin, J. Liu, S. Li, J. Liang, X. Liu, L. Xie, G. Lu, J. Han, Y. Huang, Q. Li, Anticorrosive SnS2/SnO2 heterostructured support for Pt nanoparticles enables remarkable oxygen reduction catalysis via interfacial enhancement, Adv. Funct. Mater., 2023, 33, 2211638.

20. L. Jiao, T. A. Arman, S. Hwang, J. Fonseca, N. Okolie, E. Shaaban, G. Li, E. Liu, U. Pasaogullari, S. K. Babu, S. Mukerjee, J. S. Spendelow, D. A. Cullen, F. Jaouen, Q. Jia, Self-sacrificial template synthesis of Fe–N–C catalysts with dense active sites deposited on a porous carbon network for high performance in PEMFC, Adv. Energy Mater., 2024, 14, 2303952.

21. P. Zhang, H.-C. Chen, H. Zhu, et al., Inter-site structural heterogeneity induction of single atom Fe catalysts for robust oxygen reduction, Nat. Commun., 2024, 15, 2062.

22. D. Shen, F. Sun, Z. Liang, H. Fu, L. Wang, Axial-N induced square-pyramidal crystal field of atomically iron sites for enhancing acidic oxygen reduction, Angew. Chem. Int. Ed., 2025, e202505937.

23. J. Xu, Y. Meng, X. Qiu, H. Zhong, S. Liu, L. Zhang, J. Zhang, P. Hou, S. P. Beckman, F. Wu, C. Liu, M. Shao, J. Li, Honeycomb-like single-atom catalysts with FeN3Cl sites for high-performance oxygen reduction, Adv. Powder Mater., 2025, 4, 100298.

24. J.-F. Gu, J. Wang, C. Wang, J. Li, C. Chen, N. Zhang, X.-Y. Xu, S. Chaemchuen, Two-dimensional ZIF-L derived dual Fe/FeNx sites for synergistic efficient oxygen reduction in alkaline and acid media, J. Colloid Interface Sci., 2025, 684, 159–169.

25. Y. Liu, Q. Li, Y. Liang, J. Liang, X. Sui, Z. Wang, Controlled preparation of Fe–N–C catalyst with ordered macroporous framework for superior acidic oxygen reduction performance, J. Alloys Compd., 2025, 1036, 182115.

26. Y. Liu, G. Li, M. Chen, Y. Liu, T. Qiu, X. Han, J. Li, R. Li, X. Shi, Z. Kang, Q. Pan, X. Tian, P. Rao, Curved Fe–N4 sites endow effective oxygen reduction reaction in acid medium, Chem. Eng. J., 2025, 521, 167202.

27. T. Zhou, Y. Guan, C. He, et al., Building Fe atom–cluster composite sites using a site occupation strategy to boost electrochemical oxygen reduction, Carbon Energy, 2024, 6, e477.

28. J. Li, W. Xia, X. Xu, D. Jiang, Z.-X. Cai, J. Tang, Y. Guo, X. Huang, T. Wang, J. He, B. Han, Y. Yamauchi, Sharply expanding single-atomically dispersed Fe–N active sites through bidirectional coordination for oxygen reduction, J. Am. Chem. Soc., 2023, 145, 50, 27262–27272.

29. L. J. Yuan, B. Liu, L. X. Shen, Y. K. Dai, Q. Li, C. Liu, W. Gong, X. L. Sui, Z. B. Wang, d-Orbital electron delocalization realized by axial Fe4C atomic clusters delivers high-performance Fe–N–C catalysts for oxygen reduction reaction, Adv. Mater., 2023, 35, 2305945.
